# Sustaining Problem-Based Learning (PBL) in medical programs: insights from two continents

**DOI:** 10.1186/s12909-026-10786-7

**Published:** 2026-11-10

**Authors:** Elin Karlsson, Priscah Mosol, Katarina Kågedal, Madeleine Abrant Dahlgren, Malin Lindqvist Appell, Pius Musau

**Affiliations:** 1 Department of Health, Medicine, and Caring Sciences, Linköping University, Linköping, Sweden; 2 Department of Nursing Education, Leadership Management and Research, Moi University, Eldoret, Kenya; 3 Department of Biomedical and Clinical Sciences, Linköping University, Linköping, Sweden; 4 Department of Surgery and Anaesthesiology, School of Medicine, Moi University, Eldoret, Kenya

**Keywords:** Problem-based learning, Collaborative learning, Student-centred learning, Higher education, Tutor

## Abstract

**Background:**

Problem-based learning (PBL) is a well-established approach in health-professions education, valued for promoting self-directed collaborative learning, but its implementation varies widely across contexts. Linköping University and Moi University have adopted PBL for decades despite facing challenges such as limited resources. This was a comparative study aiming to explore experience of first-year medical students and tutors regarding PBL in two universities in different continents.

**Methods:**

Four focus groups were conducted with tutors and students respectively, from Moi University and Linköping University. In total, 24 participants were included. Data was analyzed using thematic analysis.

**Results:**

A prominent main theme was identified: system support for upholding the principles of PBL. Within this theme, five subthemes were developed unravelling various understandings of the core principles of PBL, as well as practices of PBL. The role of tutors was considered crucial, although their prerequisites and systemic support were not always sufficient. The findings highlight how clarity of procedures requires solid and committed leadership on various levels for a successful PBL process.

**Conclusions:**

PBL supports active, collaborative learning, but its sustainability depends on having adequate institutional support. Without clear leadership, adequate resources, and ongoing competence development, the PBL principles risk being diluted over time.

**Supplementary Information:**

The online version contains supplementary material available at https://doi.org/10.1186/s12909-026-10786-7.

## Background

Problem-based learning (PBL) is widely recognized as a core instructional approach in health-professions education (HPE), designed to foster self-directed learning, collaborative problem-solving, and the integration of basic and clinical sciences [1–4].

Linköping University (LiU) in Sweden is often cited as an early leader in this model; since the 1980s, its Faculty of Medicine and Health Sciences has organized its curriculum around integrated, case-based themes delivered in small groups guided by trained facilitators [5–8]. This approach aims to promote early clinical exposure, continuous reasoning across disciplines, and the development of lifelong learning skills [1, 9].

At Moi University’s (MU) College of Health Sciences in Kenya, PBL has been incorporated since the late 80s [10], but more gradually, and its implementation reflects both enthusiasm and structural difficulty. Surveys of faculty show that although many lecturers value PBL for enhancing student independence, teamwork, and creativity, a substantial proportion have lacked prior training in facilitation when they joined the institution [11, 12]. Graduates of Moi University’s PBL-based programmes nonetheless report stronger leadership and self-management skills than those from traditional lecture-centered curricula [10].

Despite these benefits of PBL-based curricula, challenges remain. LiU and MU have an ongoing collaboration that has lasted for more than 30 years, leading to knowledge exchanges on pedagogical matters such as PBL, as well as research collaboration such as a recent relating work by Musau et al. [13]. Limited physical resources, large class sizes, and reliance on conventional assessment methods often hinder the full realization of PBL’s student-centered goals [2, 12]. There is a need to continuously adapt the PBL model to contemporary demands, including increased student numbers [4] as well as evolving assessment requirements on the backdrop of artificial intelligence. HPE needs to be in line with such societal changes and thus re-evaluated [14, 15]. Coherent alignment must exist between learning outcomes, PBL activities, and assessment strategies to maintain the integrity of the approach [4]. Further, literature from other contexts underscores the importance of sustained faculty development, adequate learning spaces, and deliberate curricular integration to ensure successful implementation [16].

This study is a comparative project examining perceptions and practices of PBL. Similar comparisons on how PBL is perceived and enacted at LiU and MU have been sparsely conducted, although a recent study within the same larger project has been conducted [13], although focusing on student in the latter stages of their education as opposed to the present study focusing on first year students. Cultural and social differences between the two sites must be acknowledged when comparing experiences in these two contexts. For instance, Sweden is a high-income country with a generous welfare system, including the healthcare system in which the students are intended to spend their career as future physicians. In contrast, Kenya represents a lower‑middle‑income setting where healthcare provision is shaped by resource constraints, uneven access, and substantial regional variation. These different structural conditions at the two sites influence not only how care is delivered but also how students make sense of their professional role, their responsibilities, and the limits of the system they are learning to navigate. The present study seeks to contribute knowledge on what the two contexts can learn from each other and how to move towards successful delivery of the PBL model in medical programmes, serving as inspiration and not solely focusing on comparisons.

## Aim

The aim of the study was to explore the experience of first-year medical students and tutors regarding PBL in two universities in different continents.

## Methods

### Study setting

The medical programmes at MU and LiU were both founded in the 1980s, have a curriculum fully conducted using PBL with tutorials twice a week. Both sites have, in total, approximately 800 registered medical students (MU) versus 1200 (LiU). MU has one campus in cooperation with a single teaching hospital and 16 primary care units. LiU has four campuses and cooperates with nine hospitals and 106 primary care units. Both universities each have about 100 teachers connected to the medical program, and an additional 80–100 affiliated clinical teachers. For the LiU teachers there is a mandatory PBL course for new teachers, but there is no mandatory course for the MU teachers, although it used to be. In both institutions, tutorials are conducted for two hours twice a week using a stepwise PBL model. At LiU the tutorials consist of 6–8 medical students while in MU, they are often twice the size. In both settings, the tutors are drawn from both basic and clinical sciences.

### Data collection

The participants involved in this focused group discussion were purposively selected.

At MU, the class leader (a student responsible for coordinating the academic activities) for the first term on the medical programme was informed of the project, its aims and benefit to the students, faculty and institution. She was then requested to recruit students willing to participate in a focus group. This purposive selection was such that only those interested turned up for the focus group discussion, and those who could not make it or were not interested in participating never got to be known by the researchers. Seven first-year students (four females and three males), all in their twenties, participated in the focus group interview, led by the last author, at Campus in November 2023. The discussion lasted for 67 min.

The MU tutors were recruited for the interviews by the last author and consisted of six participating tutors: five males and one female. All tutors had at least ten years experience of the role as being a tutor in a PBL group, and were either lecturers or senior lecturers, with one assistant professor. The focus group interview was conducted at Campus in August 2023 and led by the last author, together with the third and fifth author. The discussion lasted for 60 min.

At LiU, participants were recruited from a randomly selected tutorial group of first-year students. Recruitment was conducted by the third author. In total, seven first-year students (four females and three males) participated in the focus group, held at Campus in February 2023. This discussion was led by the last and third author and lasted for 60 min.

LiU tutors were approached by the third author. Four tutors participated in the focus group, two females and two males, and lastied for 65 min. Similarly as the MU tutors, all LiU tutors had at least ten years experience of the role as being a tutor in a PBL group. The majority of them were assistant professors while one was a professor. The last and third author led the discussion, held at Campus in April 2023.

The discussions in all groups followed an interview guide, developed within a larger project previously published [13]. The interview guides for students and tutors are available as supplementary material. The interview guides were focused on students’ and tutors’ perceptions of the PBL tutorial process as a teaching and learning method, their experiences with the method, the roles of tutors and students in tutorials, resources used in their learning, challenges faced and suggested ways of addressing them, and their views of PBL as a teaching and learning method. All four focus groups were audio- recorded before being transcribed verbatim.

### Data analysis

A thematic analysis was employed, which is a qualitative method used to identify recurring themes and patterns within data [17, 18]. In this study, a latent thematic analysis with an inductive orientation was conducted, following Braun and Clarke’s guidelines, to uncover underlying meanings in the material. The six steps in Braun & Clarke’s [17] thematic analysis move from familiarizing yourself with the data, to coding key features, grouping codes into themes, reviewing and refining those themes, defining and naming them, and finally producing a coherent analytic report. The dataset in this study—comprising four focus group transcripts, was initially skimmed by the first, second, and last author and discussed at an online meeting. During a subsequent on-site workshop, the first, second, and last author discussed emerging patterns and initial ideas, and a brief summary of this brainstorming process was documented. A more detailed reading followed, after which the same authors met again and together selected relevant excerpts and coded these into a central file. This was performed by these three authors together for three of the four transcripts. The fourth transcript was coded by the first author. This specific coding was read through and validated by the second and last author, who had minor suggestions that were incorporated into the central file. Subsequently, the codes were organized into preliminary themes by the first author, with both subthemes and an overarching theme identified. The central file including the preliminary themes and subthemes was scrutinized by the second and last author, who had suggestions such as splitting a particularly rich subtheme into two. The themes and subthemes were then revised accordingly and refined by clarifying their labels and content aiming to develop rich, solid themes. The final stage involved drafting the article and selecting quotes that best illustrated each theme, in accordance with Braun & Clarke [17]. The second and last authors provided feedback on the first draft of the results, leading to adjustments, after which all authors of this manuscript read and provided feedback. All authors contributed their expertise in various phases of the process. These discussions led to revisions, including changes in quote selection, condensation of material, and reorganization of themes and text.

### Ethical considerations

This study was conducted in accordance with the internationally recognized ethical principles of the Declaration of Helsinki which safeguard the rights, and well-being of research participants [19]. For instance, in this study, potential participants were provided with information about the study, that participation was voluntary, that they were free to withdraw from the study at any time without the need to provide a reason, that data would be treated with confidentiality, and that their responses would be anonymized when used in publications. Ethical approval was not required for this study, as the data collected was not considered sensitive, as assessed by the local ethics committee at MU: In the process of considering ethics for this study, The Moi University/Moi Teaching and Referral Hospital Institutional Research and Ethics Committee (IREC) was consulted to establish whether formal approval was needed for this administratively permitted evaluation of PBL experiences. Their assessment and advice were that it was deemed unnecessary but stated that informed consent from the participants was needed. This advice was complied with, and informed consent was obtained from all participants prior to the focus groups.

## Results

One main theme was identified: *System support for upholding the principles of PBL*. Within this theme, five subthemes were developed: (1) The understanding of participants of the principles of PBL, (2) The practice of PBL as a teaching and learning method, (3) The role of tutors, (4) Introducing students and new tutors to PBL and facilitating continuous competence development for tutors, and (5) Clarity of procedures requires leadership.

### The understanding of participants on the principles of PBL

In this subtheme, various views and perspectives on PBL are prominent, including the perceived significance of the method for students learning. Students highlight challenges in fully understanding PBL and sometimes “fumbling in the dark” needing guidance, as well as having a hard time deciding how much reading is enough.

*There is so much information and there is so much we don’t need to know. Sometimes it is difficult to differentiate what is important and what is not* [Students, LiU].

This is also acknowledged by the tutors as a common challenge for students in their first years. However, positive experiences of PBL are also highlighted by both tutors and students, in terms of PBL facilitating a more engaged and active learning process as opposed to solely participating in lectures. Further benefits are highlighted, such as the opportunity of gaining new perspectives through a deeper analysis of a problem together with others and having a forum to facilitate making sense of previous lectures through discussion.

*They are more engaged they are more…their mindset is a bit different they know how to seek information put it together and make sense of it* [Tutors, MU].

*…it is a matter of challenging the students to trigger their curiosity and support (them) in finding of knowledge and talking about what they learnt so they will kind of make it their own…* [Tutors, LiU].

Tutors describe how the design of scenarios for the tutorial groups are essential for shaping how the topic will be addressed as well as how PBL as a method will be understood, and that the scenarios need to spur curiosity and creativity rather than being a checklist of what to read. The clarity of scenarios is highlighted as an important aspect by the students, who have developed certain strategies to cope with uncertainty, for instance, adhering to the learning outcomes in the course outline.

*…we went through fumbling and a lot of guidance and that is why I say the course outline has really been of good help in the tutorial group that I am in*,* but sometimes they can be really tricky to get* [Students, MU].

### PBL as a teaching and learning method

This subtheme highlights variations in how PBL is adopted in the two settings. Group dynamics and feedback are central aspects highlighted by the students in terms of facilitating trust and a good working climate. Trust building and feeling comfortable together is particularly emphasized by the LiU students, but students at both sites describe that group dynamics benefit from students spending time with each other beyond the tutorial, for instance, by having lunch together. Feedback from peers at the end of a tutorial is described by students at both sites as being essential for identifying topics that were left out in the discussion and areas of improvement. Further, the tutorials commonly consist of having an assigned chairperson leading the tutorial and a designated secretary. The strategies to learn from the scenario, or case, differ. For instance, tutorial groups at MU tend to divide objectives among students that each student presents at the next tutorial, as opposed to the tutorial groups at LiU where students share the same objective and gather around it in discussions. In MU, there also seems to be a temptation among tutors to cut corners and inform the students through, for instance, giving mini lectures.

*(The period) in between is for research; studies. On the first day, you raise the objectives this other day you present so the interval is for everyone to go and read and make sure that when they come, they have something to present *[Students, MU].

*If someone is an expert in that area you may be tempted to think you are saving time by giving more information…*. [Tutors, MU].

The resources used in between tutorials span from books, YouTube-videos, lectures, to flashcards for memorization, and this in itself is considered a journey in navigating among various sources of information, finding what is most suitable for the individual. Also, students emphasize the importance of everyone in the group taking responsibility for the learning process, and that their role as students involves being prepared and helping the others in the group.

*I feel like it is sort of your responsibility to be able to contribute to each other’s collective learning and for that to be possible you need to sort of do the work. Yes. Because it is not gonna work out if a quarter of the group are not doing their fair share to help the collective learning of the group as a whole. You need to do some work in order that the process of PBL be effective. So you have this sort of personal responsibility* [Students, LiU].

### The role of tutors

The role of the tutors emerges as a key factor for adopting the principles of PBL properly, by guiding the students through the steps of the PBL process. The tutors highlight the importance of students supporting each other in PBL, and that it is not always easy for the students to distinguish what they need to learn, which leads to various survival skills being developed.

*We had learning objectives then we had the clarifications of the learning objectives and then we had the student’s collection of how to manage exams and we have kind of parallel curricula that students are creating to support and reach these learning objectives* [Teachers, LiU].

However, insufficient prerequisites for tutors to uphold the PBL principles are highlighted. For instance, despite having knowledge of PBL, the data shows how tutors use different survival skills to cope with a too heavy workload. The tutors at MU describe how the tutorials tend to consist more of the tutor giving lectures, which to some extent also occur at LiU depending on the expertise of the tutor. Tutors at MU describe how the ways to work used to be more in accordance with the PBL principles, but that this has drifted over the past years, becoming a deviation from the original ways.

*I think there are elements of it. The PBL we experienced when we were students I feel has really changed over time maybe diluted over time for various reasons they have mentioned. One is probably there is quite a number of staff who do not understand what PBL is; the second one is there are fewer staff so you find that your workload has increased and you may not have the time to do the tutorial as we used to do at that time* [Tutors, MU].

Students expect their tutors to guide them in the right direction, keeping them on track, and to be available, which is in correspondence to the perceptions of the tutors. However, at MU students describe how their tutors do not always have time to participate in the tutorial, thus, leaving them to figure it out on their own. Those who did not get adequate support were disadvantaged throughout the course and indicated that the role of the tutor faded into insignificance. Tutors also state that it is important to show an interest, to act as a role model for the students, facilitate the group dynamics, and to prepare them for their future working life.

*Because of the numbers (of staff)… initially the tutorials used to work very well but nowadays people tend, due to the workload, to give more lectures on what they are going to examine and are then done with it *[Tutors, MU].

They (the students) have to be aware of that as a tutor I am not there to provide knowledge I am there to help them find knowledg [Tutors, LiU].

### Introducing students and new tutors to PBL and facilitating continuous competence development for tutors

This subtheme highlights a desire to properly introduce students and new tutors to the principles of PBL, a matter that is considered crucial but not always managed sufficiently or consistently. The tutors at MU highlight the crucial differentiation between providing a brief overview of a topic rather than giving students a lecture, in which the latter is perceived as being clearly related to tutors who are new and/or not properly introduced.

*I think this time we are getting more of the lecture method than the PBL tutorials we used to have and I think the issue is the induction …those people don’t know what to do those who have gone through the system might be knowing what PBL is but the new staff may not be in the picture so when told overview they think it is a lecture so they lecture and go* [Tutors, MU].

Tutors at both sites state that there is a need for tutors to meet and discuss the tutorial process, learning from each other and exchanging experiences, but that a forum for this is either absent (at MU) or primarily attracting the experienced tutors rather than the new ones (at LiU).

*And I think at the beginning of every course, that should happen, tutors, course coordinators and year coordinators should meet for the introduction of the course. I think that doesn’t happen nowadays. We get the materials in our pigeon holes…the course started two weeks ago (Laughter) *[Tutors, MU].

*There is a growing group of teachers that come with the experience of the traditional way of teaching and they have kind of integrated this way of teaching …so it is very difficult to change the way you think about learning and we don’t really have a very powerful discussion in the faculty about this. We had the Problem Based Learning workshops once a semester and I would also join them and really find it worthwhile and interesting because it gives more power in the function but that is only 10% of the teachers…* [Tutors, LiU].

Another topic highlighted is the use of postgraduate students, where the tutors describe a desire for mentorship as part of teacher succession.

*Other departments that have postgraduates if they have tutorials and they cannot be able to attend they actually have been sending their postgraduate students, so it is happening informally but we need just to formalize it. I don’t know whether it will work but I think even in clinical areas we have postgraduates having some classes* [Tutors, MU].

*Our tutor is very busy so he gave us a co-tutor who is a postgraduate student who is always with us *[Students, MU].

The introduction of students is described as essential, in particular when the tutor is inexperienced, which provides a structure for both parts and facilitates reasonable expectations. However, the introduction for students has been gradually shortened at MU, which concerns the tutors. The lack of a clear introduction is also highlighted among the students.

*I would just like to tell the school to explain better the benefits of PBL to new students because at first I didn’t see the importance of PBL in fact I said why are we not being given lectures and we go and do research on our own? Right now I understand the benefits of PBL and SDL but in first term I didn’t grasp the whole concept* [Students, MU].

### Clarity of procedures requires leadership

Tutors at both sites describe how a clear structure and leadership are necessary in order for the tutors to work towards the same goal and to maintain unity in procedures. The perception is that the faculty need to provide solid leadership in line with the PBL principles, including a commitment to these.

Both groups of tutors highlight various aspects of organizational challenges. But depending on the site, the characteristics of these challenges are somewhat different. The LiU tutors have overarching concerns regarding the design of learning activities, specifically the exams, while the MU tutors have several practical and structural issues that affect both the tutors working conditions as well as the students learning. Tutors at MU highlight the crucial role of leadership in terms of regulating the number of students in each tutorial group, which at MU can be 15–20 or even more than thirty. This is considered challenging for the tutors to manage, and they also describe how favorable group dynamics and active participation among the students are prominent challenges, risking negative effects on students’ learning. Further aspects highlighted by the MU tutors are insufficient resources in terms of staffing, administrative support regarding scheduling classes, appropriate rooms for the tutorials, and technical equipment. For instance, the rooms allocated in the schedule for these large tutorial groups are not always big enough, leading to the tutor and students spending time from the tutorial on searching for another place to sit. These aspects are directed towards the university management, and a call for change is prompted.

*And then also the timetable … the daily timetable is violated I don’t think anybody follows them so it only brings confusion. You are waiting for students to come for a tutorial they are sitting an exam elsewhere (Laughter) *[Tutors, MU].

*You know initially the focus of the department of medical education was really to assist everyone else to understand PBL and implement it properly but along the way there seems to have been a shift in their focus which is not a bad thing but you see it means that the other aspect and priority has gone down now they are focusing on running their academic programmes* [Tutors, MU].

The tutors at LiU highlight how the exams are not fully aligned with the PBL principles due to the increased usage of multiple-choice questions. They state that the questions that they actually would want the students to answer are often too much work to manage.

*So we have an exam system that forces us into questions we can manage to correct and that also is sort of a problem and that then reflects on the faculty and the way we look at knowledge of how we administer the exams* [Tutors, LiU].

## Discussion

This study aimed to explore the experience of junior students and tutors with PBL in two different countries on two continents. The findings demonstrate that students and tutors alike describe both challenges and benefits of PBL, such as difficulties for first-year students in discerning relevant knowledge but also recognizing its capacity to foster active and engaged learning. Group dynamics and feedback are emphasized as crucial, though practices vary between the two settings. Tutors play a pivotal role in guiding students through the PBL process, yet at MU heavy workloads and insufficient resources often dilute PBL into lecture-based teaching. The introduction of new students and tutors to PBL is essential for avoiding misunderstandings and inconsistent application of its principles. Opportunities for tutors to exchange experiences and develop competence vary between the settings, where such forums are absent at MU while at LiU they primarily attract the more experienced tutors rather than the new ones, which ultimately is weakening the sustainability of PBL practices. There seem to be challenges in the continuous education of new teachers, particularly in maintaining engagement with PBL and ensuring it remains a vital part of the faculties’ practices. Leadership promoting PBL and clear organizational structures are viewed as essential to ensure coherence and alignment with PBL principles. At MU, large tutorial groups and logistical constraints pose significant challenges, which has also been demonstrated in other studies [13]. In both settings, the importance of adequate resources, effective leadership, commitment to PBL principles, and continuous competence development is highlighted. Thus, sustaining PBL requires systemic support, for both tutors and students.

In designing collaborative learning activities, it is essential to distinguish between learning in a group and learning as a group, the latter of which is more beneficial for students learning outcomes in PBL-based education [20–22]. For instance, MU students divide a broader topic into individual sub‑topics, with each student preparing their own presentation. Although efficient, this structure does not require collaboration or shared meaning‑making as the group functions as a set of individuals. Learning as a group, by contrast, involves students jointly engaging with a shared problem that cannot be meaningfully divided. LiU students who work together to solve a common task exemplify this approach. Here, progress depends on interaction, negotiation, and collective reasoning. Group-worthy tasks and PBL scenarios should preferably spur curiosity and promote teamwork. If a problem can be divided into topics that individual students can solve, then it is not a group-worthy task. Further, although students sometimes may prefer traditional, passive lectures as demonstrated elsewhere [2, 4], there is a need to ensure a balance in teaching and learning by integrating complementary lectures that reinforce rather than undermine PBL [4]. Tutors’ passion for lecturing and students’ expectations can unintentionally shift the curriculum toward a teacher‑centred design, making it essential to recognise this tension in order to preserve the principles of PBL [4].

In order to sustain the principles of PBL in medical education, leadership and system-level support play a crucial role. Clear leadership and solid support structures have the potential to facilitate unity and clarity in the tutorials, beneficial for both students and tutors. Previous research highlights barriers including inadequate resources such as space allocation [2, 12], shortage of tutors, lack of appropriate technology and equipment [12]. The prerequisites for the tutors to deliver support to students have been demonstrated as essential in other studies [22, 23], for instance in terms of ensuring sufficient competence for the tasks [23]. Ensuring competence and continuous competence development requires strong systemic support. Yet, despite broad advocacy for more and better forums, the time and resource investments required to sustain such initiatives often generate organizational resistance to development [4]. Similarly as stated by Musau et al. [13], a reintroduction of a PBL-course for tutors at MU could facilitate reflection and feedback on the PBL procedures. This is in line with Ge et al. [24], stating that the homogenization of tutors must be put back on the agenda. Other studies have pointed towards potential solutions in terms of integrating technology more as a way of supporting tutors and students as well as facilitating equal opportunities for learning [25]. A PBL curriculum may be perceived as demanding in terms of time and resources compared to more traditional curricula, which calls for sufficient numbers of tutors, adequate equipment and relevant competence development [12]. In this study, the MU tutors used various coping strategies to manage an excessively heavy workload, leading to a drift in the usage of PBL. The role of leadership and adequate resources in making PBL a success is eminently prominent.

### Methodological considerations

The study findings should be considered in light of some methodological limitations. A qualitative study’s trustworthiness should be evaluated in terms of transferability, credibility, dependability and confirmability [26]. First, purposeful sampling may pose challenges to the transferability of the findings as the participants represent a small percentage of the target population, and this recruitment strategy potentially could lead to a homogenous sample of participants. Further, in research, there is always a risk that those who choose to participate are the most interested in the subject, the most critical, or the most satisfied. The risk of selection bias should thus be considered. Worth noting is the usage of different sampling strategies at the two universities, which potentially could lead to variations in experiences or attitudes, relating to the sampling procedure. However, no significant differences that clearly related to this were prominent in the focus groups.

In this study, we chose the two settings and the qualitative methodology in order to delve deeper into the research questions and to share insights from two rather experienced universities, rather aiming for providing inspiration and sharing lessons learned. Thus, although the sample is small, the findings can hopefully provide inspiration and insight to others working with PBL in medical programs. The analysis was carefully conducted through validation of several of the authors. EK, PM and PiM did the majority of the coding together, leading to discussions and unity, before EK continued with the latter analytical steps, which were discussed and validated again. This is a strength of this study, that provided findings through triangulation and a systematic approach. Further, while coding, EK, PM and PiM discussed contextual terms and events described by the participants, which were all clarified as all are experienced teachers working with PBL and either of the two universities. This preunderstanding of the topic should be noted as it could entail difficulties for an objective interpretation of the data. Because the coding mainly was conducted together, such risks were minimized and instead a richer understanding of the context of both sites was obtained.

## Conclusions

This study underscores that while PBL fosters active engagement and collaborative knowledge construction, its sustainability hinges on robust systemic support. Effective leadership, adequate resources, and structured opportunities for continuous competence development are essential to ensure coherence and fidelity to the PBL principles. Without such institutional commitment, challenges such as diluted practices, uneven tutor preparation, the usage of survival skills among tutors to cope with a too heavy workload, and logistical constraints risk undermining its long-term viability across diverse educational contexts. Future research should focus on how to achieve such institutional commitment and how to sustain it over time.

## Supplementary Information

Supplementary Material 1.

Supplementary Material 2.

## Data Availability

The data generated in the current study is not available, because the participants have consented to participate in studies conducted by this research group, and thus, we do not have their consent to share the data.
