# Supplementary material for: Sustaining Problem-Based Learning (PBL) in medical programs: insights from two continents
Source: BMC Med Educ. 2026 Nov 10;26:1830. doi: 10.1186/s12909-026-10786-7 (PMC13753190; doi:10.1186/s12909-026-10786-7)
Supplement: Supplementary file 1 [file 12909_2026_10786_MOESM1_ESM.docx]

PBL EVALUATION PROJECT IN LINKOPING AND MOI UNIVERSITIES

**GUIDE FOR THE STUDENT FOCUS GROUP DISCUSSIONS**

The interaction in the FGDs will reflect the ingredients of the PBL process: the problem; resources; objectives; behaviour and skills; learning that is interactive and active; exemplification of higher order thinking; motivation; and self as well as group reflection and evaluation.

QUESTIONS

1. **Tutorial group, size and its appropriateness for PBL as well as the collaborative behaviour of the students**

-Is a tutorial group important for learning in PBL?

*This should bring out the interactive aspect, individual contribution to the group learning and ease of managing the group dynamics when people are few.*

-What happens in a tutorial group?

*The organization with team leader, the scribe, active brainstorming of issues, critical and analytical thinking to elicit learning issues and acquisition of skills like communication and teamwork.*

2. **The co-ownership of the learning process and the respective roles of the tutors and students**

-What is your role as a student in the PBL process?

*The student as the centre of the learning process with responsibility for learning*

- What is the role of the tutor in the PBL process?

*The tutor is seen as a guide, mentor or facilitator of the learning process.*

-In what ways do you help each other while learning through PBL?

*They bring in their past knowledge and individual experiences, leverage on their individual traits and learning styles and collaborate to yield the maximal learning for the group.*

3. **The problem and objectives**

-How would you describe the PBL scenario concerning the learning process?

*The problem should be ill-structured, real-life and open-ended enough to allow for a wide exploration of the learning field in order to facilitate multidisciplinary and integrative learning. It should not be solved as an end to itself but to aid in the learning.*

-In your experience, can the learning issues (lärandemål) be derived from the PBL scenario without additional information from the tutor?

*This will give an idea as to the quality of the tutorial problems in relation to the expected standards of the ideal PBL.*

4. **Resources and reflection at end of PBL session**

-What resources do you rely on to make the PBL successful?

*The students will outline the various activities they undertake and the resources available. These may include library and varied materials, expert inputs during SDL, laboratory work, large group lectures and simulation sessions.*

-Do you consider these resources adequate?

*This will inform us on the challenges the students may be encountering in their learning.*

-What is the importance of reflection and feedback in the PBL process?

*This should bring forth the relevance of assessing the process, individuals and the group with regard to the activities during the tutorial so as to benefit each other as well as improve collectively where necessary.*

-Are you sufficiently motivated by the PBL as a learning method?

*The reflective phase in which the students will evaluate the PBL, their roles and that of the tutor and whether the whole process synergizes the learning to a point of finding it a worthy method of learning.*

-What else would you like to say regarding PBL as a learning method?

*The students will give their views on PBL as a learning method beyond what has been asked and thus tie the loose ends in the discussion.*

**= End =**
