# Supplementary material for: Sustaining Problem-Based Learning (PBL) in medical programs: insights from two continents
Source: BMC Med Educ. 2026 Nov 10;26:1830. doi: 10.1186/s12909-026-10786-7 (PMC13753190; doi:10.1186/s12909-026-10786-7)
Supplement: Supplementary file 2 [file 12909_2026_10786_MOESM2_ESM.docx]

PBL EVALUATION PROJECT IN LINKOPING AND MOI UNIVERSITIES

**TUTOR FOCUS GROUP GUIDE**

The focus group discussion will aim at establishing the tutors’ years of teaching experience , the method of learning they themselves went through in their formative (undergraduate) years, possible biases towards the two methods of teaching, their considered roles in PBL and how they view the state of PBL as presently practiced in the two institutions.

1. **Tutors’ training, experience and biases.**

The traditional lecture method and PBL have been in existence for quite a while. In what ways would they influence each other?

-*This should bring out how some parts of the PBL can conveniently be handled through lectures or large group sessions, personal biases based on their training or circumstance, how their years with PBL have refined their roles as tutors and whether they have preferences of a method over the other.*

*-The question should bring forth the role of our method of learning and its influence on how we teach particularly if one has not had any training in being a facilitator in the PBL process.*

2. **Tutor’s roles in the PBL process**

What is a tutor’s role in the PBL teaching method?

- *This question will trigger a discussion of the primary role of a tutor as a facilitator, guide, mentor and co-owner of the process with the students.*
- *It should also enable the group to enumerate the other aspects of PBL where the tutor can be assigned a lecture or overview to enhance the learning or be consulted as an expert in a given aspect of the problem.*

3. **How the PBL process is perceived to be currently**

In your own assessment, what would you say is the state of PBL here based on your experiences?

- *This will lead to a discussion on the process of PBL, its requirements and whether the tutors consider what is practised to be close to or different from the ideal PBL.*

4. **Suggestions towards improving PBL**

Where there are serious issues in need of correction to align PBL with the ideal practice, what corrective measures would you suggest?

- *This should bring out the key areas of deficiency be it staff and their training, size of tutorial groups, formulation of the problems, adequacy of resources or promotion of motivated lifelong learning.*

**=End =**
